# miR-107 is involved in the regulation of NEDD9-mediated invasion and metastasis in breast cancer

**DOI:** 10.1186/s12885-022-09603-3

**Published:** 2022-05-12

**Authors:** Jiamin Zhou, Xianglin Sun, Xinyu Zhang, Huan Yang, Zhenglin Jiang, Qianqian Luo, Yifei Liu, Guohua Wang

**Affiliations:** 1 Department of Physiology and Hypoxic Biomedicine, Institute of Special Environmental Medicine, Nantong University, 9 Seyuan Road, Chongchuan District, Nantong, 226019 Jiangsu China; 2 Department of Pathology, Affiliated Hospital of Nantong University, 20 Xisi Road, Nantong, 226001 China

**Keywords:** Breast cancer, miR-107, NEDD9, Cancer metastasis, Cancer invasion

## Abstract

**Background:**

As a metastasis-related protein, NEDD9 has been reported in breast cancer (BC) metastasis research. However, there are few studies on the upstream regulators of NEDD9, especially involving the potential role of miRNAs. The purpose of this study was to explain whether miR-107 potentially regulates NEDD9, which may lead to invasion and metastasis of BC.

**Methods:**

MCF-7 and MDA-MB-231 cells were transduced with lentiviruses to construct stably transduced cells with miR-107 overexpression, miR-107 silencing or empty vectors. A luciferase reporter assay was performed to verify the binding of miR-107 and NEDD9. The scratch test and Transwell assay were used to measure cell migration and invasion ability, respectively. For the study of metastasis in vivo, we injected MDA-MB-231 cells into the fat pad of nude mice to develop an orthotopic breast cancer model.

**Results:**

We found that NEDD9 expression correlates with the prognosis of BC patients. In BC cell lines, NEDD9 was positively correlated with cell migration ability. Further research revealed that miR-107 inhibited NEDD9 expression by targeting the 3′-untranslated region of NEDD9. Overexpression of miR-107 suppressed the expression of NEDD9, thereby inhibiting the invasion, migration and proliferation of BC cells, but interference with miR-107 promoted the expression of NEDD9 as well as invasion, migration and proliferation. In an in vivo model, overexpression of miR-107 decreased the expression of NEDD9 and inhibited tumour growth, invasion and metastasis; however, these effects were reversed by inhibiting miR-107.

**Conclusions:**

These findings indicated the potential role of miR-107 in regulating NEDD9 in the invasion, migration and proliferation of BC.

**Supplementary Information:**

The online version contains supplementary material available at 10.1186/s12885-022-09603-3.

## Background

Breast cancer (BC) is the most common cancer in women worldwide [1, 2]. In general, primary BC is not fatal; however, metastatic BC is generally considered incurable [3]. The uncontrolled proliferation of BC epithelial cells results in malignant mutations and then in metastases to lymph nodes or other organs, which is a highly lethal reason for BC patients [4]. Therefore, prevention and treatment of BC metastasis are particularly important in the treatment of BC [5]. For this reason, there is an urgent demand to better comprehend the molecular mechanism of BC metastasis, which will help to identify new biomarkers that can be used to predict metastasis and as therapeutic targets for the treatment of BC patients with metastasis [6].

The NEDD9 (also known as HEF1 and CASL) protein belongs to the Crk-associated substrate (CAS) family of linker molecules [7, 8]. The NEDD9 protein does not have any known enzymatic functions but contains a domain that interacts with a variety of functional proteins, which play a significant role in the molecular signalling pathways related to tumour metastasis [8, 9]. Previous studies have also shown that NEDD9 is a key molecule that promotes BC progression by promoting migration and invasion [10, 11]; however, there are few studies on the upstream regulators of NEDD9, especially involving the potential role of microRNAs (miRNAs).

miRNAs are small noncoding RNAs composed of 17–25 nucleotides, and it is well known that miRNAs modulate gene expression in eukaryotic cells [12]. MiRNAs target the 3′-untranslated region (3′-UTR) of mRNAs that are involved in cellular processes in different species [12, 13]. It is recognized that altered expression of miRNAs participates in the occurrence, development and metastasis of tumours by aiming at the tumour suppressor genes or mRNAs of oncogenes [14–16]. In addition, various kinds of cancer cells have revealed completely different miRNA expression profiles, suggesting that the analysis of miRNA expression patterns may help identify miRNAs that modulate tumour progression [17, 18]. miRNAs are becoming more established as cancer regulatory molecules; however, the role of miRNA expression in BC development and the potential of miRNAs as markers for diagnosis, prognosis and pharmacogenomics still need to be determined [18, 19].

In this work, the binding between miR-107 and the 3′-UTR of NEDD9 was demonstrated by bioinformatics prediction and dual-luciferase reporter gene assays [20]. We found that NEDD9 was significantly upregulated in BC and had a direct correlation with the poor prognosis of patients. The qRT-PCR and western blotting results suggested that miR-107 regulates the expression of NEDD9. In vitro experiments showed that the overexpression of miR-107 restrained the proliferation, invasion and migration of BC cells by inhibiting NEDD9. In vivo experiments confirmed that the invasion and metastasis of BC were suppressed after miR-107 overexpression. Overall, the present results indicated that miR-107/NEDD9 might be a new molecular target for the prevention and treatment of BC metastasis.

## Methods

### Cell lines

Wild-type (WT) human MCF-7, MDA-MB-468 and MDA-MB-231 BC cell lines were obtained from the Institute of Biochemistry and Cell Biology, Chinese Academy of Sciences (Shanghai, China). Short tandem repeat (STR) profiling data were obtained to demonstrate that the MCF-7, MDA-MB-468 and MDA-MB-231 cells were derived from the appropriate parent cell line by Biowing Applied Biotechnology Co., Ltd. (Shanghai, China). All cells were cultured in humidified air containing 5% CO_2_ at 37 °C. MCF-7 cells were cultured in minimum essential medium (MEM, Gibco, Waltham, USA) with 10% foetal bovine serum (FBS, Gibco, Waltham, USA), and MDA-MB-231 cells were cultured in Dulbecco’s modified Eagle’s medium (DMEM, Gibco, Waltham, USA) with 10% FBS. All cell lines were freshly thawed every 2 months and used within 20 passages.

### Construction of stable cell lines

MCF-7 and MDA-MB-231 cells in the logarithmic phase in a 6-well plate were transfected with a miR-107 interference lentivirus (miR-107-shRNA) or NC-shRNA, a miR-107 overexpression plasmid (miR-107-OE) or an empty plasmid, which were all purchased from OBiO Technology, Corp., Ltd. (Shanghai). The multiplicity of infection (MOI) for lentivirus was 20, and the MOI for the plasmids was 10. Polybrene was operated at a final concentration of 5 μg/mL, and the medium was changed 24 h after transfection. Forty-eight hours after transfection, 1 μg/mL puromycin was added for selection. The medium was replaced every 2–3 days, and the cells were passaged after 5–6 days. After 4 passages, the remaining cells were considered stably transfected cells. The expression of miR-107 was calculated by qPCR to verify the success of the transfection.

### RNA extraction and qPCR

Total RNA was collected from cells with TRIzol reagent, and the miRcute Enhanced miRNA cDNA First Strand Synthesis Kit (Tiangen, Beijing, KR211) was used for reverse transcription [21]. The miRcute Enhanced miRNA Fluorescence Quantitative Detection Kit (Tiangen, Beijing, FP411) was utilized to detect the expression of miR-107 for qPCR, and U6 was employed as the internal reference. The primers were as follows: upstream primer of miR-107, AGCAGCAUUGUACAGGGCUAUCA; and upstream primer of U6, CTCGCTTCGGCAGCACA. The downstream primers were provided by the kit.

### Western blot

Total protein was extracted in lysis buffer with protease inhibitors. After proteins were separated on a 10% SDS-PAGE gel, they were transferred to a cellulose acetate membrane. After blocking with 5% skim milk, the membrane was incubated with NEDD9 (1:1000, ab18056, Abcam, USA) and β-actin (1:10000, A5316, Sigma, USA) antibodies at 4 °C overnight. The membrane was subsequently washed and incubated with a goat anti-mouse secondary antibody (1:10,000, 115–035-003, Jackson, USA) for 2 h at room temperature. After the membrane was fully washed, chemiluminescence was performed for the development and visualization of the protein bands.

### Luciferase reporter assay

To predict the miRNAs that regulate NEDD9, we used the TargetScan [22], DIANA and MiRanda databases [23]. The bioinformatics raw data has been uploaded as supplementary materials. The target reporter plasmid containing the 3′-untranslated region (3′-UTR) of the 3′-UTR of mutant NEDD9 or WT was used for the target luciferase reporter assay. A total of 1 × 10^4^ HEK-293 T cells (American Type Culture Collection, Manassas, VA, USA) were seeded in a volume of 200 μl in each well in 96-well plates. According to a previous method [24], a total of 100 ng of WT or mutant reporter gene construct was cotransfected with 50 nM miR-107 mimic or miR-NC into HEK-293 T cells with Lipofectamine 2000 transfection reagents. After 48 h, luciferase activity was detected with a dual luciferase reporter gene detection kit (E1910, Promega, USA). Relative luciferase activity was calibrated to firefly luciferase activity.

### Cell proliferation

Cell proliferation was assessed by a colony formation assay. A total of 5000 stably transfected cells were seeded in each well of a 6-well plate. The cells were cultured for 7–14 days, and the medium was changed every 3–4 days. Colony formation was evaluated under a microscope. Each single cell that proliferates in vitro more than six times will form a single colony. After the formation of colonies, the medium was discarded, and the cells were washed gently with PBS two to three times. Cells were stained with crystal violet for 30 min, and the number of single clones was counted to determine cell proliferation ability.

### Cell migration and invasion analysis

A scratch test was carried out to measure cell migration ability according to the protocol in our laboratory [25]. The abovementioned stably transfected cells were plated in a 6-well plate. After the cells covered the bottom of the 6-well plate, a sterile 100-μl pipette tip was utlized to scrape the cell monolayer. The cells that were scraped off were washed with PBS and cultured in serum-free medium, and the area of the wound was determined. After 24 h of culture in a 37 °C incubator containing 5% CO_2_, the wound area was measured again. Cells cultured under serum-free conditions for 24 h were regarded as cells that migrated without proliferating, and the wound area reflected the migration ability of the cells. In the cell invasion experiment, the above stably transfected cells were added to the upper layer of a Transwell migration chamber (pore size, 8 μm; Corning, 3422) covered with Matrigel (356,234, Biocoat, USA) in medium with 1% foetal bovine serum. The lower chamber was added to medium with 10% foetal bovine serum, and the plate was incubated in a 37 °C incubator containing 5% CO_2_ for 24 h. MDA-MB-231 cells and stably transfected MDA-MB-231 cells were cultured for 12 h, and the bottom of the upper chamber was wiped with a cotton swab. The nonmigrated cells were then fixed with methanol for 30 min, stained with Giemsa for 30 min and counted under a microscope.

### Animal experiments

Forty BALB/c background female nude mice (6–8 weeks) were obtained from Shanghai Experimental Animal Centre of Chinese Academy of Sciences. All experiments involving animals were performed in accordance with Nantong University’s laboratory animal management regulations and were approved by the Research Ethics Committee of the Institute of Nantong University (protocol #NT-18-019). The guidelines for animal anaesthesia and euthanasia were performed in accordance with the National Institutes of Health’s Guide for the Care and Use of Laboratory Animals [26]. Animal suffering was minimized to the greatest extent possible. For straightforward monitoring of tumor growth, WT MDA-MB-231 cells and stably transfected miR-107-shRNA, NC-shRNA, miR-107-OE and NC-OE cells were subcutaneously inoculated into the left dorsal side of BALB/c mice (*n* = 5 in each group). Mice were weighed every 3 days, and the longest diameter and shortest diameter of each tumour were measured with a Vernier calliper. The following formula was used to measure the tumour volume: V = a × b^2^/2, where a is the longest diameter, and b is the shortest diameter. When tumours approximated 1000–1500 mm^3^, the mice were anesthetized by intraperitoneal injection of 40 mg/kg sodium pentobarbital (Cat# P-010, Millipore Sigma) for tumour removal, and tumours were collected for subsequent histochemistry and other experiments.

To observe the inhibitory effect of miR-107 on tumour growth and lung metastasis, GFP-lentivirus transduced MDA-MB-231 cells were xenografted into nude mice by surgical orthotopic transplantation. MDA-MB-231-GFP, −miR-107-OE and -NC-OE stable transgenic cells were selected and injected into the fat pad of BALB/c nude mice at a concentration of 5 × 10^6^ (*n* = 5 in each group). Mice were weighed weekly, and the longest and shortest tumour diameters were measured with Vernier callipers. The tumour volume was calculated, and lung metastasis was observed by a small animal IVIS imaging system (Lumina II, Calliper Life Sciences) as described in previous work of our institute [27, 28]. The general illumination settings and image acquisition parameters were used as suggested by the IVIS system, and fluorescence intensity in regions of interest (ROIs) was measured by the software [27, 28].

### Immunohistochemistry

Paraffin-embedded sections were deparaffinized and hydrated, and antigen retrieval was performed with sodium citrate. Sections were blocked with 5% donkey serum at 30 °C for 1 h, incubated with mouse monoclonal anti-NEDD9 (Mouse, 1:1000, Cat# ab18056, RRID: AB_10851607, Abcam) at 4 °C overnight and washed 3 times with TBST buffer. Sections were then incubated with HRP-conjugated goat anti-mouse secondary antibody, washed 3 times with TBST and stained according to the instructions of the DAB staining kit (DS-0005, Zhongshan Jinqiao, Beijing). Nuclei were stained with haematoxylin (Beyotime, Jiangsu, China). After the reaction was stopped with tap water, sections were sealed with neutral gum (Beyotime). All sections were visualized under a DM4000B microscope (Leica Microsystems, Wetzlar, Germany) at a final magnification of × 200, and analyzed with ImageJ software (National Institutes of Health, Bethesda, MD, USA).

NEDD9-positive results were defined as cells containing evenly stained yellow or brown cytoplasmic granules. A scoring system combining the proportion of immunoreactive cells with the intensity of immunostaining was used to evaluate immunoreactivity, as previously described [29]. Specifically, expression of NEDD9 was evaluated according to the staining intensity and percentage of positive-stained cells. The intensity of NEDD9 staining was scored as follows: 0 (no staining), 1 (weak staining), 2 (moderate staining), and 3 (strong staining). The percentage of stained tumor cells on each section was counted and scored as follows: 0 (less than 5%), 1 (5–25%), 2 (26–50%), and 3 (more than 50%) accordingly. The scores of each case were multiplied to give a final score of 0, 1, 2, 3, 4, 6, or 9. The scores 0 to 3 were regarded as low NEDD9 expression and more than 3 as high expression.

### Statistical analysis

SPSS version 16.0 (SPSS, Inc., Chicago, IL, USA) was applied for statistical analysis. After the homogeneity of variance test, two-tailed Student’s t-test was selected to measure differences between the two groups. One-way analysis of variance (ANOVA) was performed to compare the mean results for greater than two groups. The rates of overall survival were calculated using the Kaplan-Meier method. The data are shown as the mean ± standard error of the mean (SEM). *P* < 0.05 was considered significantly different.

## Results

### High expression of NEDD9 is positively correlated with poor prognosis in BC patients

**Fig. 1:**
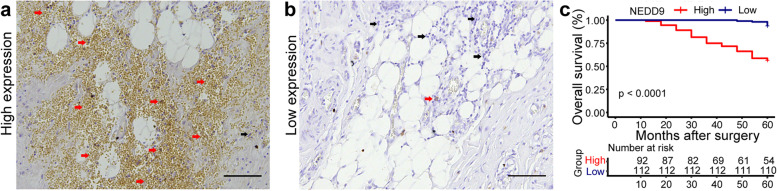
NEDD9 expression is negatively associated with the prognosis of BC patients. **a** Breast cancer patients with high NEDD9 expression (red arrow, stained yellow or brown cytoplasmic granules). **b** Breast cancer patients with low NEDD9 expression (black arrow, stained blue). **c** Kaplan-Meier survival curve illustrating the significance of NEDD9 expression in BC patients. *n* = 112 (low NEDD9 expression); *n* = 92 (high NEDD9 expression). Scale bar = 100 μm

We collected prognostic data from 204 breast cancer patients in Affiliated Hospital of Nantong University (92 patients with high NEDD9 expression and 112 patients with low NEDD9 expression). The relations between NEDD9 and clinicopathological features and molecular subtyping of breast cancer were analyzed. In 204 breast cancer patients, NEDD9 expression was significantly correlated with tumor grade (χ^2^ = 4.311, *P* = 0.038), ER (estrogen receptor) (χ^2^ = 11.16, *P* = 0.001), PR (progesterone receptor) (χ^2^ = 4.026, *P* = 0.045), Ki67 (χ^2^ = 6.558, *P* = 0.01) and lymphatic metastasis (χ^2^ = 14.79, *P* = 0.0001). However, there was no significant relation between NEDD9 and age, tumor size, HER2 (human epidermal growth factor receptor 2) and TNM (Table 1). Combination with the histopathological staining results of NEDD9, it was found that the survival rate of patients with high NEDD9 expression (Fig. 1a) was significantly lower than that of patients with low NEDD9 expression (Fig. 1b), indicating that NEDD9 expression is associated with the metastasis and prognosis of BC patients (Fig. 1c, *P* < 0.001).

**Table 1:** Relations between NEDD9 and clinicopathological parameters of breast cancer

Group	Number of cases	NEDD9		χ2	***P*** value
		Low	High		
**Age (years)**				0.033	0.984
≤ 40	18	10	8		
40 ~ 60	123	68	55		
≥ 60	63	34	29		
**Grading of tumors**				4.311	0.038
I ~ II	167	86	81		
III	37	26	11		
**Tumor size (cm**^**3**^**)**				2.059	0.151
I ≤ 2	100	60	40		
I > 2	104	52	52		
**ER (Estrogen receptor)**				11.16	0.001
Negative	95	64	31		
Positive	107	48	61		
**PR (Progesterone receptor)**				4.026	0.045
Negative	141	84	57		
Positive	63	28	35		
**HER2 (Human epidermal growth factor receptor 2)**				0.018	0.892
Negative	161	88	73		
Positive	43	24	19		
**Ki67**				6.558	0.01
Low	100	64	36		
High	104	48	56		
**Lymphatic metastasis**				14.79	0.0001
N0	133	60	73		
N1 + 2 + 3	71	52	19		
**TNM**				4.791	0.091
I	55	22	33		
II	107	60	47		
III	42	30	12

### NEDD9 is highly expressed in BC cells with strong migration ability

**Fig. 2:**
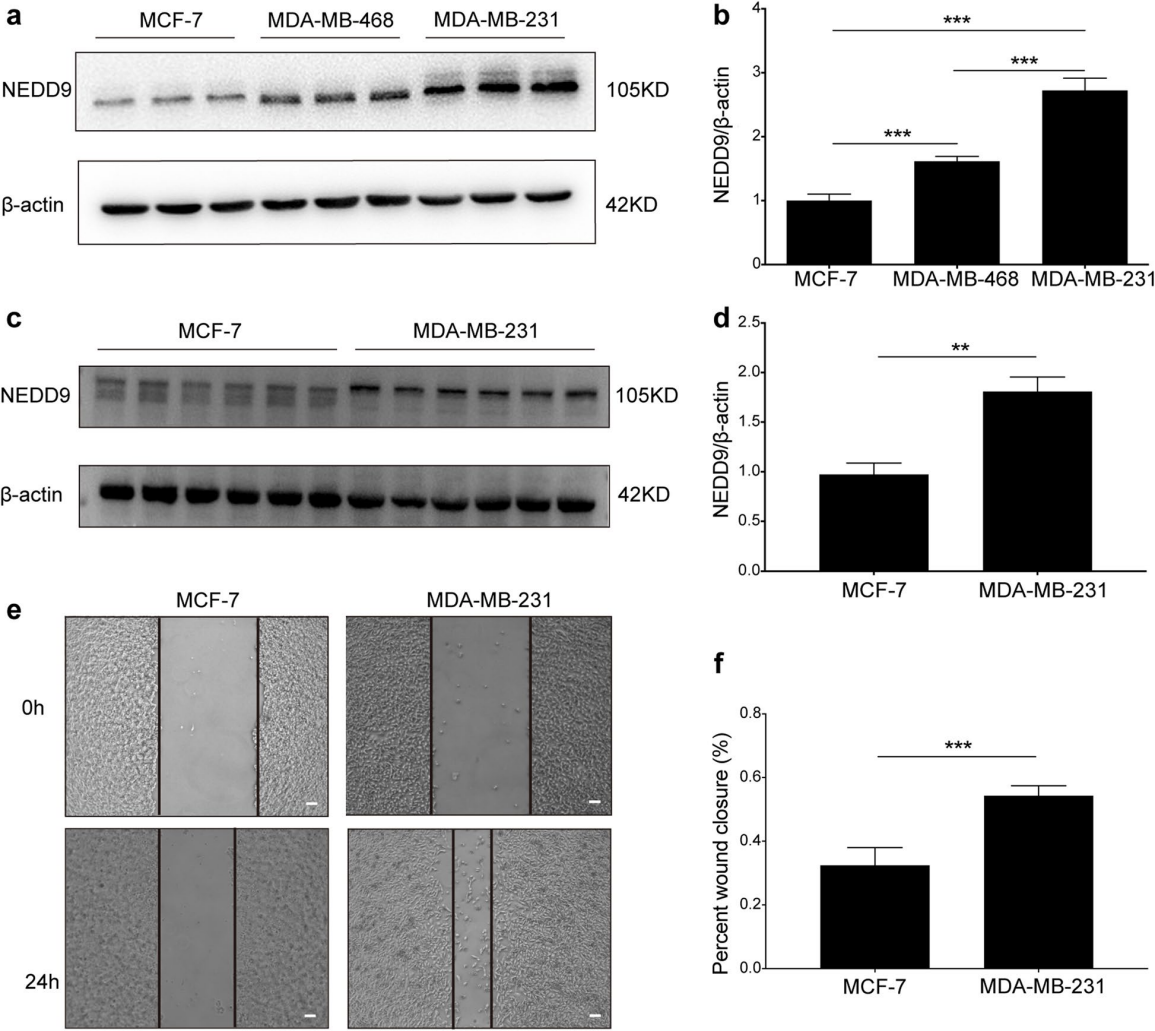
NEDD9 is highly expressed in BC cells with strong migration ability. **a** Western blot analysis of NEDD9 expression in MCF-7, MDA-MB-468 and MDA-MB-231 cell lines. **b** Statistical analysis of NEDD9 expression in MCF-7, MDA-MB-468 and MDA-MB-231 cell lines (*n* = 3). Scale bar = 100 μm. **c** Western blotting was used to measure the expression of NEDD9 in cells 24 h after the cell scratch test. **d** Western blotting was used to measure the expression of NEDD9 in cells 24 h after the cell scratch test (*n* = 6).**e** Representative images of the 24-h wound-healing ability of MCF-7 and MDA-MB-231 cells. **f** Statistical analysis of wound-healing ability (*n* = 6). ***P < 0.001, **P < 0.01

We assessed the expression of NEDD9 in MCF-7, MDA-MB-468 and MDA-MB-231 cells and showed that NEDD9 was differentially expressed in the three cell lines (Fig. 2a and b, *P* < 0.001). We further selected the low-expressing MCF-7 cell line and the high-expressing MDA-MB-231 cell line (Fig. 2c and d, *P* < 0.01) and assessed the migration ability of the two cell lines. By evaluating the wound closure changes after 24 h in the cell scratch test, we found the migration ability of MDA-MB-231 was significantly higher than MCF-7 (Fig. 2e and f, *P* < 0.001). These results suggested that the migration ability may be due to the increased expression of NEDD9.

### MiR-107 may regulate the expression of NEDD9

**Fig. 3:**
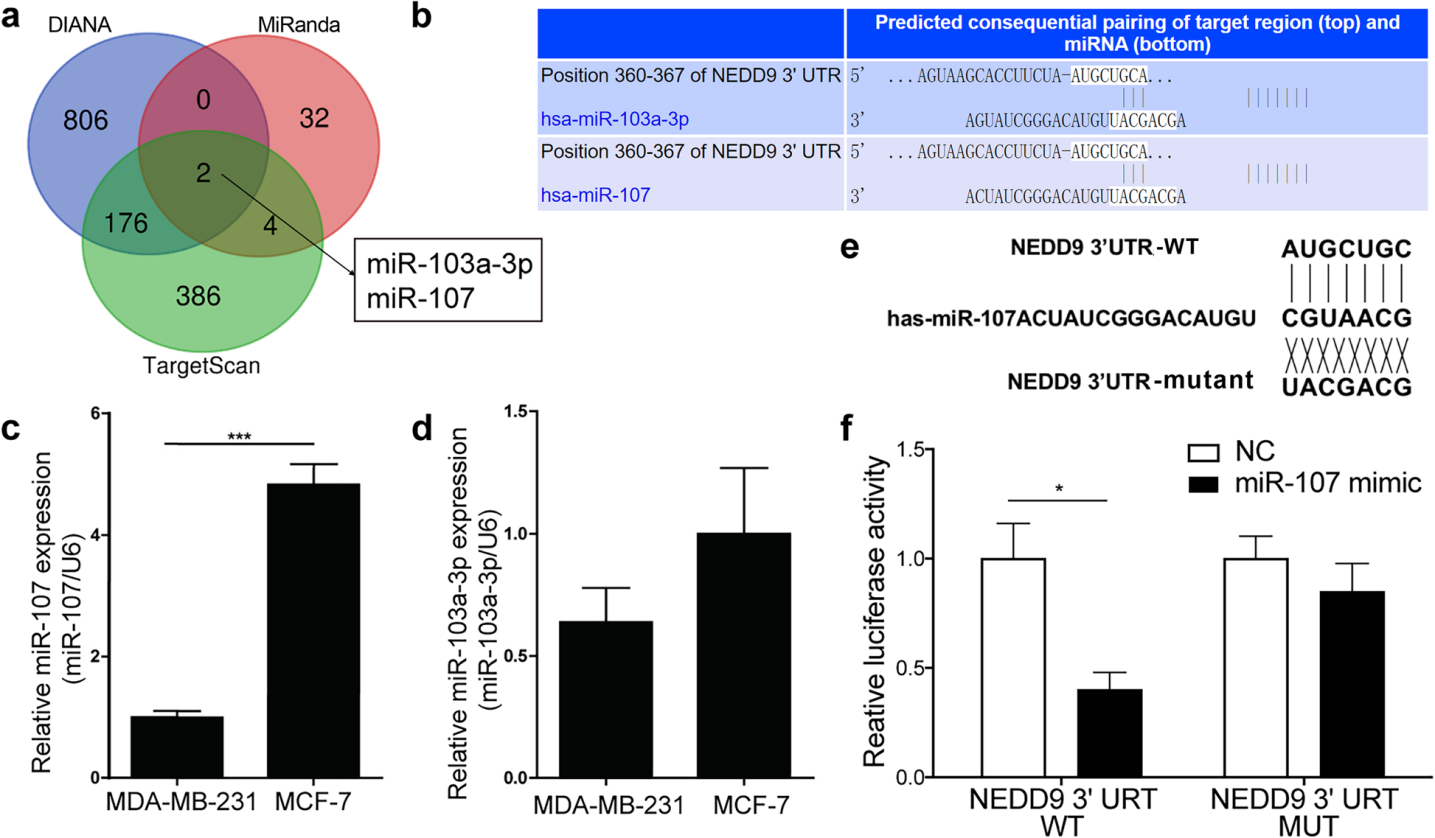
MiR-107 modulates the expression of NEDD9. **a** Common miRNAs predicted by TargetScan, DIANA and miRanda to regulate NEDD9. **b** TargetScan software was used to predict the binding sites of miR-103a-3p, miR-107 and NEDD9. **c** qPCR analysis of the expression of miR-107 and miR-103a-3p in MCF-7 and MDA-MB-231 cells. However, only miR-107 was differentially expressed. **d** Construction of fluorescein-expressing wild-type NEDD9 plasmids and fluorescein-expressing plasmids encoding NEDD9 with mutations at the miR-107-binding site. **e and f** Transfection of the fluorescein-expressing wild-type NEDD9 plasmids and fluorescein-containing plasmids encoding NEDD9 with mutations at the miR-107-binding site, miR-107 mimic and NC mimic into 293 T cells. Binding was detected based on luciferase activity (*n* = 3). ***P < 0.001, *P < 0.05

**Fig. 4:**
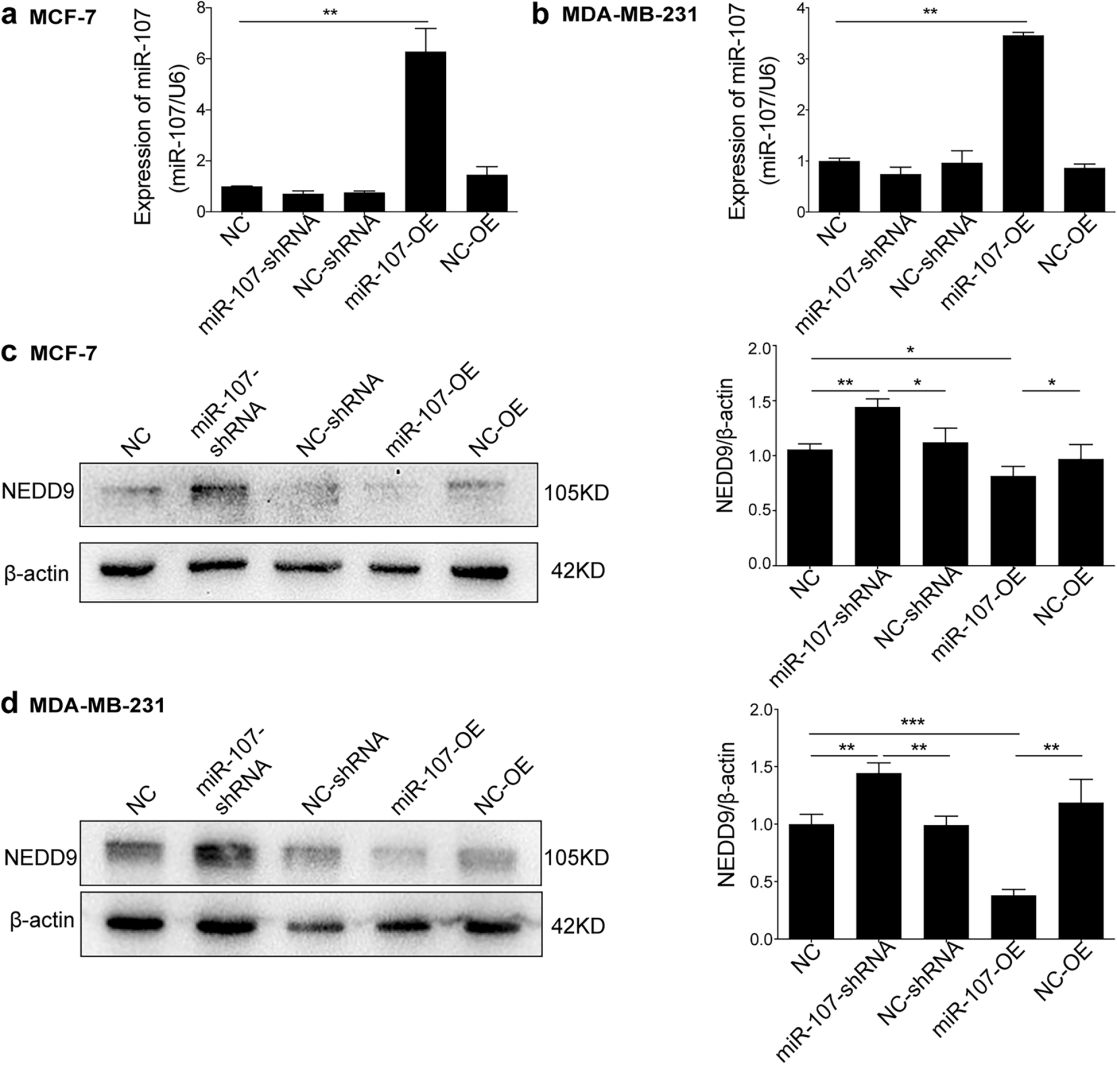
MiR-107 inhibits NEDD9 expression in BC cells. Stably transfected lines overexpressing miR-107 or silencing miR-107 were constructed. MCF-7 (**a**) and MDA-MB-231 cells (**b**) were also transfected with an empty vector. The expression level of miR-107 in the stably transfected cell lines was detected by qPCR. Western blotting was performed to assess the overexpression and silencing of miR-107 in the stably transfected lines and the expression of NEDD9 in empty vector-transfected MCF-7 (**c**) and MDA-MB-231 cells (**d**). *n* = 6; ***P < 0.001, **P < 0.01, *P < 0.05

All three databases (TargetScan, DIANA and MiRanda) predicted that miR-107 and miR-103a-3p might be involved in regulating NEDD9 (Fig. 3a, Supplementary Table 1). Because NEDD9 expression is differential in MCF-7 and MDA-MB-231 cells, they have different migration abilities. The differentially expressed miRNAs in these two cell lines may be important for the regulation of migration. We observed the expression of miR-107 and miR-103a-3p in MCF-7 cells and two strains of MDA-MB-231 cells. It was found that only miR-107 was differentially expressed in the two cell lines and that the expression of miR-107 in highly metastatic MDA-MB-231 cells was lower than that in MCF-7 cells with migration ability (Fig. 3c, *P* < 0.001), indicating that miR-107 may regulate NEDD9 in BC cells. According to the prediction made by TargetScan software, miR-107 may bind to the 3’UTR of NEDD9 at the position shown in Fig. 3b. For this reason, we constructed WT luciferase reporter gene plasmids and plasmids with mutations at this position (Fig. 3d). We cotransfected these plasmids with miR-107 mimic and NC mimic into 293 T cells and found that miR-107 decreased the luciferase activity of the WT NEDD9 3’UTR, while the mutant and mimic controls had no effect (Fig. 3e, f). After miR-107 was overexpressed by lentivirus in MCF-7 and MDA-MB-231 cells (Fig. 4a and b), the expression of NEDD9 was inhibited; however, the expression of NEDD9 was elevated significantly in MCF-7 and MDA-MB-231 cells after interference with miR-107 (Fig. 4c and d*, P* < 0.05 or < 0.01). These results showed that miR-107 is associated with regulating the expression of NEDD9 in BC cells.

### MiR-107 is associated with BC cell metastasis and proliferation

**Fig. 5:**
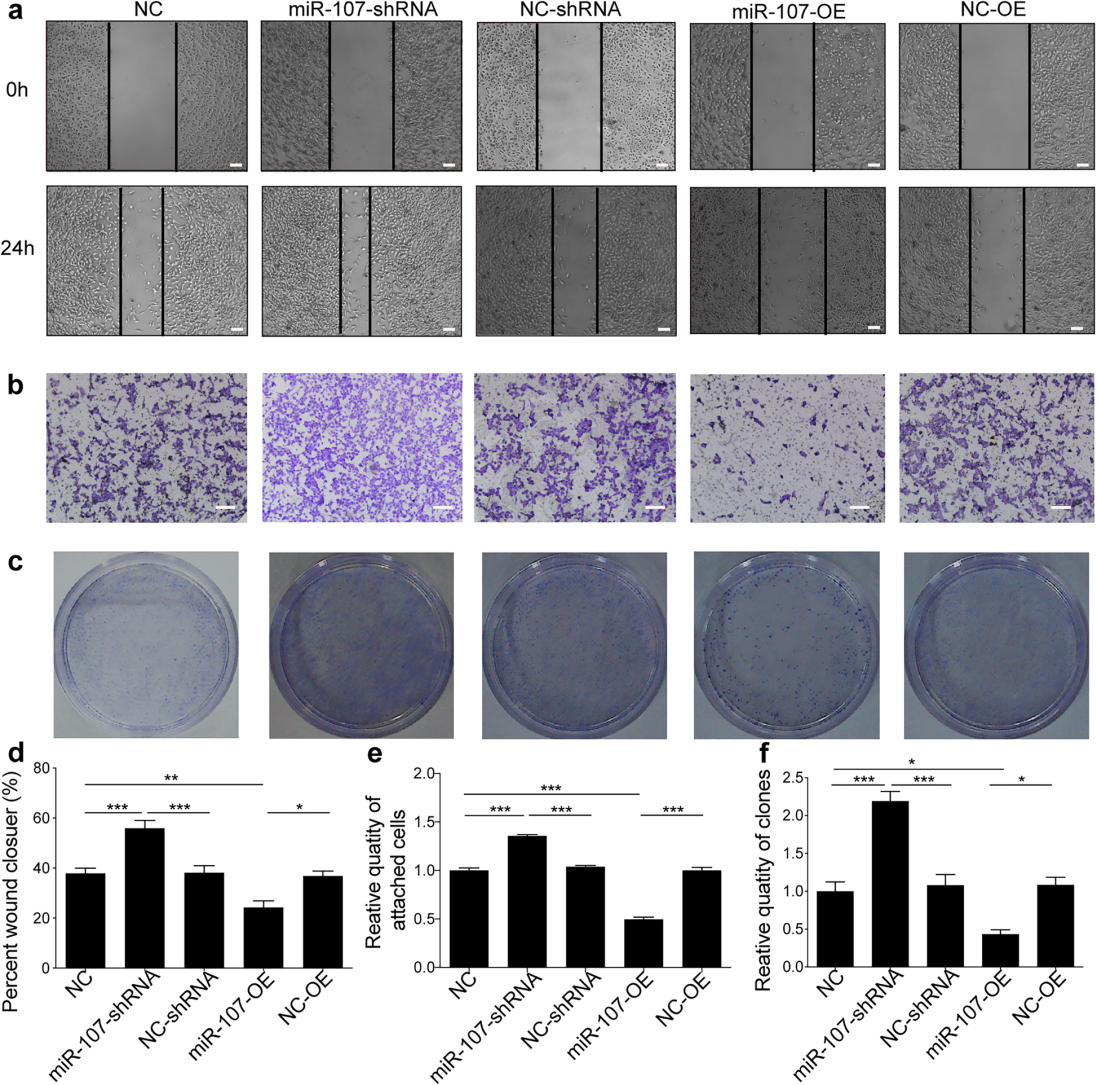
MiR-107 ipromotes the migration, invasion and proliferation of MCF-7 cells. **a** The scratch test was used to assess the migration ability of stably transfected lines with miR-107 overexpression and miR-107 silencing as well as of empty vector-transfected cells. Scale bar = 100 μm. **b** Transwell assays were used to assess the migration ability of cells with miR-107 overexpression and miR-107 silencing as well as of empty vector-transfected MCF-7 cells. Scale bar = 100 μm. **c** The colony formation assay was used to evaluate the proliferation ability of stably transfected cells with miR-107 overexpression and miR-107 silencing as well as cells transfected with empty vector. ***p < 0.001, **p < 0.01, *p < 0.05. **d** Statistical analysis of the wound closure ability (*n* = 6). **e** Statistical analysis of the migration ability of cells (*n* = 6). **F** Statistical analysis of the proliferation ability of cells (*n* = 6)

Because NEDD9 is a protein related to BC metastasis, miR-107 might also be involved in cell metastasis. After interference with miR-107, the wound-healing ability of MCF-7 cells was higher than that of untreated cells and empty vector-transfected cells (Fig. 5a, d; *P* < 0.05 or *P* < 0.001). However, once miR-107 was overexpressed, the wound-healing ability of MCF-7 cells was lower than that of untreated cells and empty vector-transfected cells (Fig. 5a, d; *P* < 0.01).

Invasion, which is different from metastasis, is the ability to infiltrate and break through the matrix at the primary site, and it was mainly evaluated by the Transwell assay. After inhibiting the expression of miR-107, the number of two types of tumour cells that passed through the Transwell membrane increased (Fig. 5b, e; *P* < 0.001), indicating that interference with miR-107 may promote the invasion ability of BC cells. When miR-107 was overexpressed, the number of cells that passed through the membrane was lower than that in the untreated group and the empty vector-transfected group (Fig. 5b, e; *P* < 0.001).

A colony formation assay was performed to verify the change in cell proliferation ability. The ability of the two cell types to form colonies following interference with miR-107 was greater than that of cells in the empty vector-transfected group (Fig. 5c, f; *P* < 0.01). When miR-107 was overexpressed, the ability of tumour cells to form colonies was lower than that of the cells in the control and empty vector-transfected groups (Fig. 5c, f; *P* < 0.05 or *P* < 0.001), indicating that miR-107 is involved in inhibiting tumour proliferation. Similar results of metastasis (Fig. S1a, d), invasion (Fig. S1b, e) and colony formation (Fig. S1c, f) were found in MDA-MB-231 cells.

### MiR-107 participates in the growth, invasion and metastasis of BC by regulating NEDD9 in vivo

**Fig. 6:**
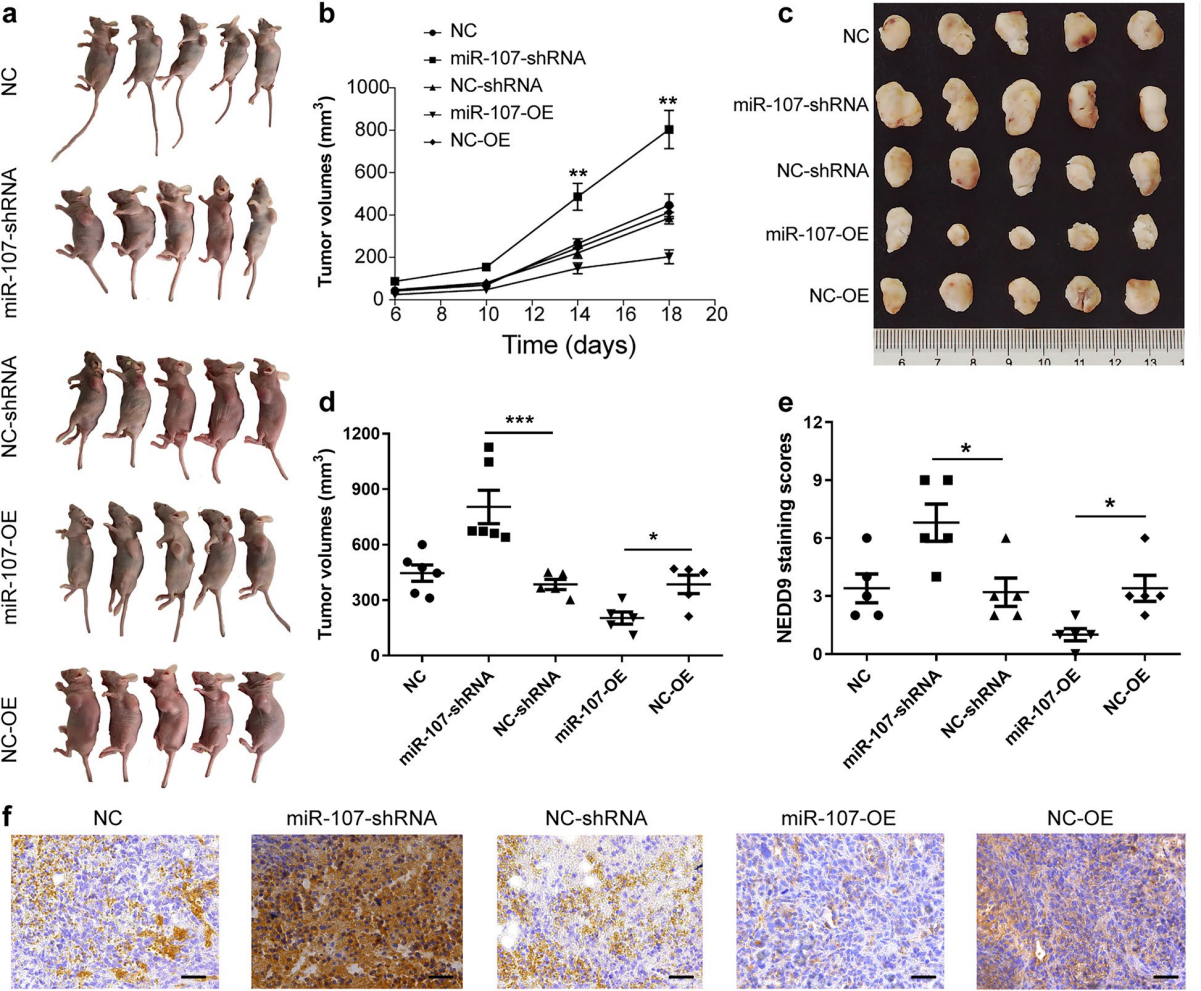
MiR-107 affects BC progression by regulating the expression of NEDD9 in vivo. Nude mice were subcutaneously inoculated with stably transfected MDA-MB-231 cells with miR-107 overexpression or silencing. After the tumours were observed, tumour volume and mouse body weight were measured every 4 days. **a** Diagram of subcutaneous tumour formation in nude mice. **b** Tumour growth curve. **c, d** Tumour volume of xenograft tumours in different groups. **e, f** Scoring analysis and representative immunohistochemical images of NEDD9 expression in xenograft tumours (scale bar = 50 μm). *n* = 5; ***p < 0.001, **p < 0.01, *p < 0.05

To study the role of miR-107 in vivo, nude mice were subcutaneously inoculated with stably transfected miR-107-overexpressing or miR-107-silenced MDA-MB-231 cells. The tumour growth rate of mice inoculated with MDA-MB-231 cells overexpressing miR-107 was lower than that of mice in the other groups, while the tumour growth rate of mice inoculated with miR-107 silencing was higher than that of mice in the other groups (Fig. 6a and b; *P* < 0.01). However, there was no difference in body weight among the groups (data was not shown). At 19 days after subcutaneous injection of MDA-MB-231 cells, the tumours of mice from each group were removed to confirm the inhibitory effect of miR-107 on tumour growth in vivo (Fig. 6c and d; *P* < 0.05 or *P* < 0.001). Immunohistochemical staining revealed that NEDD9 was inhibited in tumours overexpressing miR-107, while the opposite effect was observed in tumours with miR-107 silencing (Fig. 6e and f*; P* < 0.05). Thus, miR-107 may control tumour progression by inhibiting the expression of NEDD9 in vivo.

**Fig. 7:**
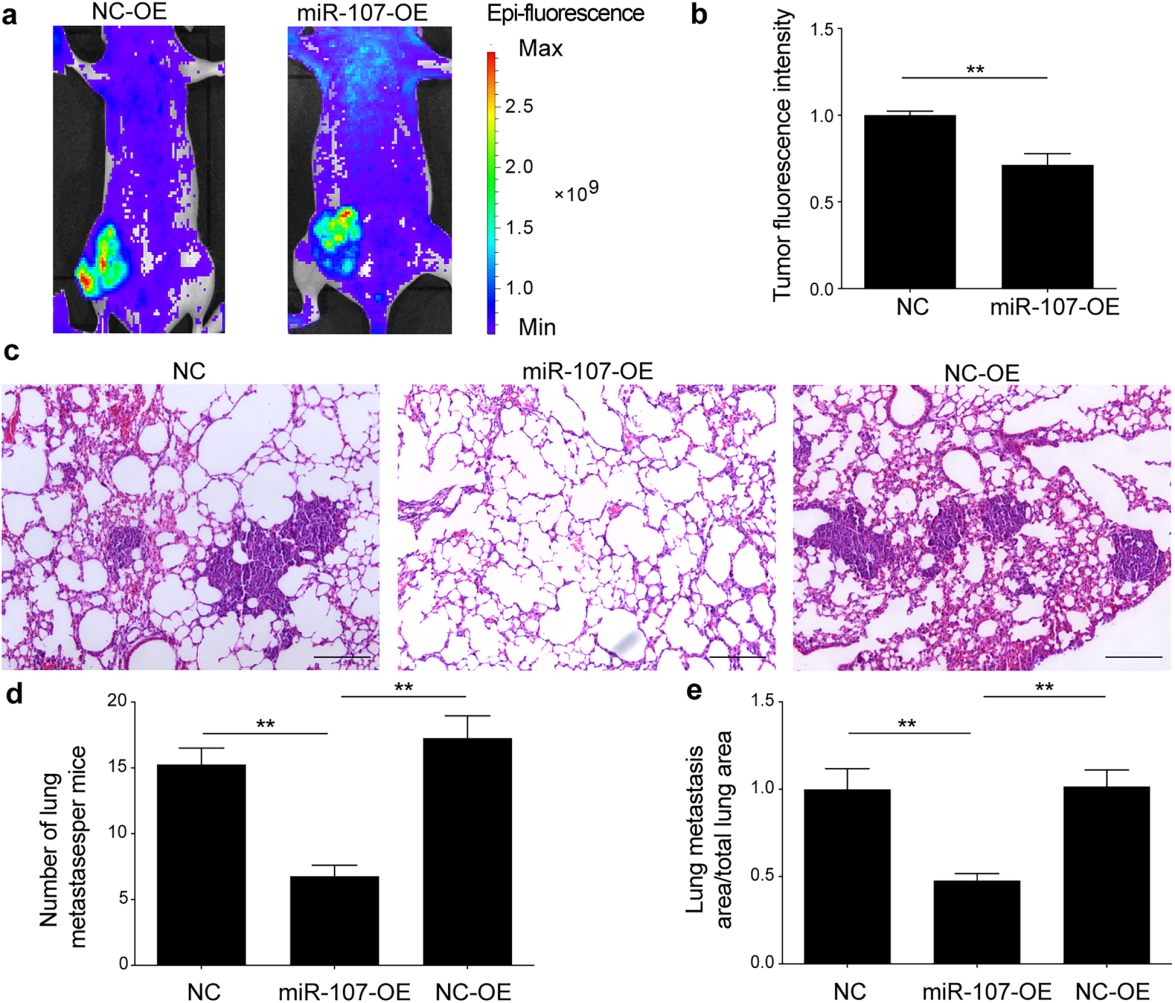
miR-107 contributes to the inhibition of breast cancer metastasis in an orthotopic breast cancer model. **a** Bioluminescence imaging was performed at 4 weeks after inoculation. **b** Statistics of the fluorescence intensity of lung metastases in nude mice. **c** Pulmonary metastatic nodules were observed using HE staining. Scale bar = 100 μm. **d** Number of pulmonary metastatic nodules in each group. **e** Proportion of lung metastasis area compared to total lung area in each group. NC, control group; miR-107-OE, miR-107 overexpression group; NC-OE, miR-107 empty group. *n* = 5; **p < 0.01

To further verify the involvement of miR-107 in the inhibition of BC metastasis, we constructed an orthotopic breast metastasis model by injecting MDA-MB-231 cells stably overexpressing miR-107 or empty vector into the breast fat pads of nude mice (Fig. S2a). The body weight (Fig. S2b) and tumour size (Fig. S2c, d) were monitored. The results showed that there was no significant difference in body weight between the groups (*P* > 0.05). The tumour growth rate of the miR-107 overexpression group was significantly lower than that of the other groups (Fig. S2d; *P* < 0.01). Furthermore, the miR-107 overexpression and empty vector lentiviruses were cotransduced with the GFP lentivirus, and metastasis in nude cells was observed by a small animal IVIS imaging system. The fluorescence signal intensity of miR-107-overexpressing cells was not only lower at the primary site, but also lower at other body parts compared to the empty vector group (Fig. 7a, b; *P* < 0.01), indicating that miR-107 may inhibit metastasis of breast cancer in vivo. Considering that the most common metastatic sites of tumours are the bone, liver and lung, lung metastasis was further observed. The number and area of pulmonary metastatic nodules in the miR-107 overexpression group were less than those in the other two groups (Fig. 7c, b and e; *P* < 0.01).

## Discussion

BC is one of the most common malignant tumours in women [30], and the main factor leading to the poor prognosis of BC patients is the distant metastasis of cancer cells [31]. Therefore, controlling the metastasis is important for the treatment of BC [32, 33]. Various studies have shown that NEDD9 is highly expressed in malignant tumour tissues, such as glioma, colorectal and liver cancer tissues, and it has been suggested that it plays an important role in tumour invasion, metastasis and migration [34, 35]. NEDD9 is a molecule related to tumour invasion and metastasis [34, 35]. There have been several reports about the involvement of NEDD9 in the development of BC [36, 37], but the precise molecular mechanism of NEDD9 in BC metastasis remains unclear. Here, we found that the expression level of NEDD9 in BC patients was negatively related to their prognosis. The survival rate of BC patients with high expression of NEDD9 was lower than that of patients with low expression. Moreover, the expression of NEDD9 in MDA-MB-231 cells with strong migration ability was higher than that in MCF-7 cells with low migration ability. These results suggested that NEDD9 is correlated with the metastasis of breast cancer.

MiRNAs are involved in almost all aspects of cancer biology, including angiogenesis, drug resistance, apoptosis, proliferation, invasion and metastasis [38, 39]. Whether a miRNA participates in tumour suppression or carcinogenesis depends on the pathway or gene involved in tumour regulation [40]. Currently, many miRNA targets have been computationally predicted, prior to applying experimental approaches that allow a better functional characterization of miRNAs in biological processes and their potential effects [22, 41]. Based on the role of miRNAs in tumour development and the regulation of mRNAs [42], we used bioinformatics databases, including miRwalk, miRdb and TargetScan (Supplementary Table 2), to predict the upstream miRNAs that may regulate NEDD9, and we selected miR-103a-3p and miR-107 as candidates. Because NEDD9 is differentially expressed in MCF-7 and MDA-MB-231 cells, the miRNA regulating NEDD9 should also be differentially expressed in these two cell lines. qPCR experiments were further used to measure the expression of miR-107 and miR-103a-3p in the two cell lines, and only miR-107 was differentially expressed in the two cell lines. Thus, miR-107 was designated as the target miRNA of this experiment. Generally, miRNAs are associated with posttranscriptional regulation and inhibit expression by binding to the 3’UTR of target mRNAs. We constructed mutant and wild-type NEDD9 luciferase plasmids containing the predicted binding site of miR-107 and demonstrated that miR-107 inhibits the expression of NEDD9 by regulating its 3’UTR.

We further studied the role of miR-107 in regulating NEDD9 in BC metastasis. We observed that NEDD9 was highly expressed in BC cells with high migration ability, while miR-107 was expressed at a low level in BC cells with extreme invasiveness. Moreover, it was shown that miR-107 affected the proliferation ability of BC cells through colony formation assays. For the in vivo experiment, we first utilized a simple and successful subcutaneous tumour model. We inoculated untreated wild-type, miR-107-overexpressing, miR-107-silenced and empty vector-transduced MDA-MB-231 cells under the skin of nude mice. The growth rate of tumours in the miR-107 overexpression group was faster than that of the other groups, while that of the miR-107 overexpression group was slower than that of the other groups. After immunohistological staining, we further found that the expression of NEDD9 in the miR-107 group was less than that in the other groups, while NEDD9 expression in the miR-107 interference group was higher than that in the other groups. These subcutaneous tumour models indicated that miR-107 may participate in the development of breast cancer by regulating NEDD9 in vivo. Considering that the main purpose of our experiments was to explore the mechanism of metastasis in BC, we further established a metastatic model of BC that easily formed primary metastases. By injecting untreated wild-type MDA-MB-231 cells loaded with miR-107-overexpressing and miR-107-silencing vectors into the fat pad of female nude mice, spontaneous metastasis was observed. In the overexpression group, the signal was weaker than that in the empty vector group, and the signal in the mouse thoracic cavity was also weaker than that in the empty vector group. These findings showed that overexpression of miR-107 not only affects proliferation, but also inhibits the metastasis of BC cells in vivo. These in vivo and in vitro experiments showed that interfering with the expression of miR-107 alters cell migration and invasion ability and affects the expression of NEDD9; that is, miR-107 contributes to the invasion and migration of BC cells by regulating NEDD9. Thus, miR-107 plays a major role in the development of BC. We will consider miR-107 for in-depth research in the future.

In summary, our research demonstrated that the regulation of NEDD9 by miR-107 is an important step in the process of BC metastasis and that miR-107 may play a significant role in the treatment and prognosis of BC.

## Supplementary Information

**Additional file 1: Supplemental Figure S1.** MiR-107 is involved in promoting the migration, invasion and proliferation of MDA-MB-231 cells. (a) The scratch test was used to assess the migration ability of stably transfected lines with miR-107 overexpression and miR-107 silencing as well as of empty vector-transfected cells. Scale bar = 100 μm. (b) Transwell assays were selected to assess the migration ability of cells with miR-107 overexpression and miR-107 silencing as well as of empty vector-transfected MCF-7 cells. Scale bar = 100 μm. (c) The colony formation assay was used to evaluate the proliferation ability of stably transfected cells with miR-107 overexpression and miR-107 silencing as well as cells transfected with empty vector. ***p < 0.001, **p < 0.01, *p < 0.05. (d) Statistical analysis of wound closure ability (*n* = 6). (e) Statistical analysis of the migration ability of cells (*n* = 6). (f) Statistical analysis of the proliferation ability of cells (*n* = 6).

**Additional file 2: Supplemental Figure S2.** miR-107 contributes to the inhibition of breast cancer development in an orthotopic breast cancer model. (a) Stably transfected MDA-MB-231 cells overexpressing miR-107 were injected into subcutaneous mammary fat pads of nude mice. Diagram of subcutaneous tumour formation in nude mice. (b) Body weight curve. (c, d) Tumour volume of xenograft tumours in different groups. NC, control group; miR-107-OE, miR-107 overexpression group; NC-OE, miR-107 empty group. *n* = 5; **p < 0.01.

**Additional file 3: Supplemental Table S1.** The database for miRNA sequence-based prediction.

**Additional file 4: Supplemental Table S2.** Description of the parameters and values shown in the results delivered by TargetScan, miRanda and Diana Tools.

**Additional file 5.**

## Data Availability

The original data and STR profiling data presented in this study can be found in online repositories (https://www.jianguoyun.com/p/Dc0Y-RsQlvWCChioqZwE).
