# Supplementary figures and images for: miR-107 is involved in the regulation of NEDD9-mediated invasion and metastasis in breast cancer
Source: BMC Cancer. 2022 May 12;22:533. doi: 10.1186/s12885-022-09603-3 (PMC9097419; doi:10.1186/s12885-022-09603-3)

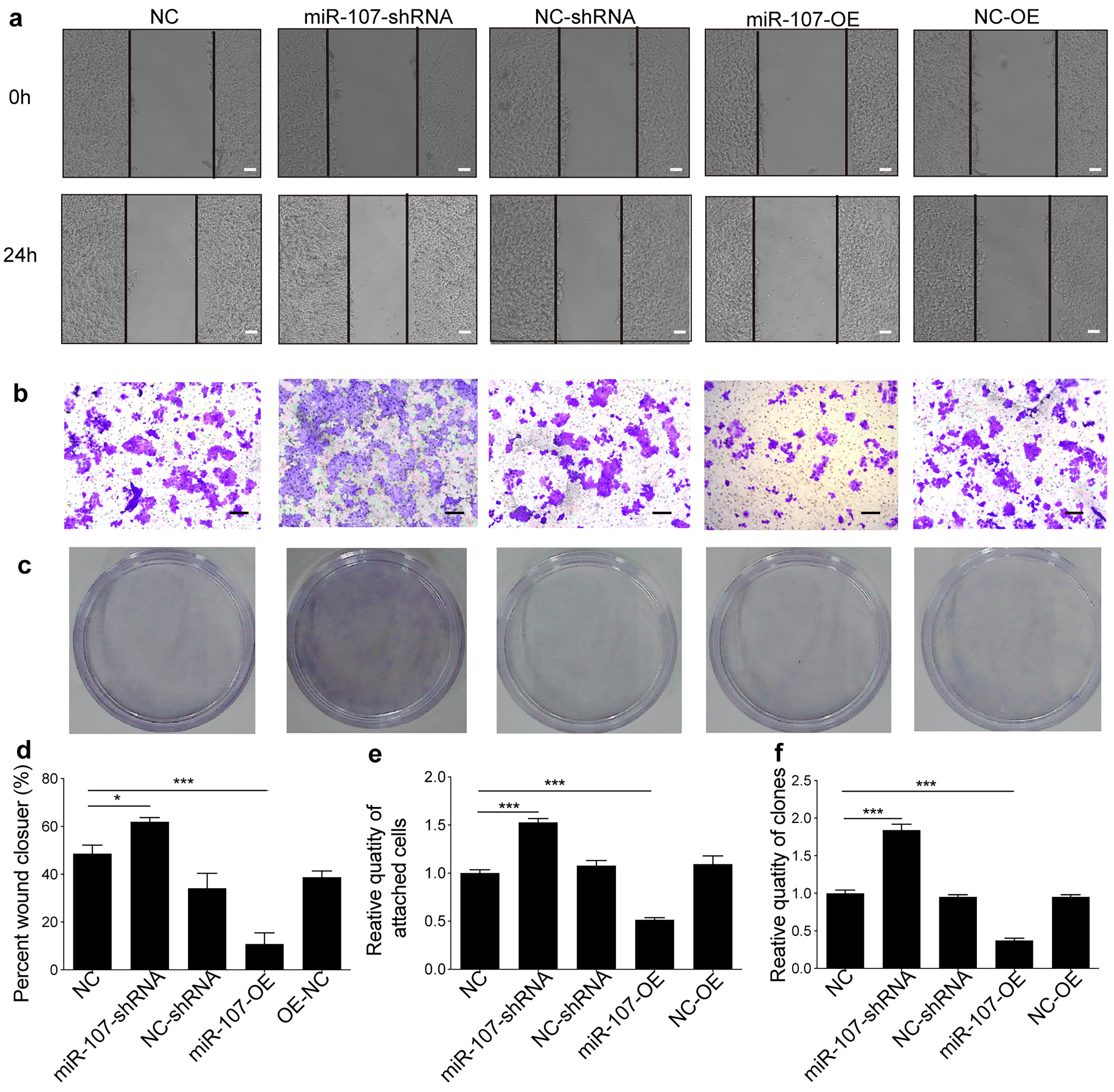

Supplement: Supplementary file 1 — Additional file 1: Supplemental Figure S1. MiR-107 is involved in promoting the migration, invasion and proliferation of MDA-MB-231 cells. (a) The scratch test was used to assess the migration ability of stably transfected lines with miR-107 overexpression and miR-107 silencing as well as of empty vector-transfected cells. Scale bar = 100 μm. (b) Transwell assays were selected to assess the migration ability of cells with miR-107 overexpression and miR-107 silencing as well as of empty vector-transfected MCF-7 cells. Scale bar = 100 μm. (c) The colony formation assay was used to evaluate the proliferation ability of stably transfected cells with miR-107 overexpression and miR-107 silencing as well as cells transfected with empty vector. ***p < 0.001, **p < 0.01, *p < 0.05. (d) Statistical analysis of wound closure ability (n = 6). (e) Statistical analysis of the migration ability of cells (n = 6). (f) Statistical analysis of the proliferation ability of cells (n = 6). [file 12885_2022_9603_MOESM1_ESM.tif]

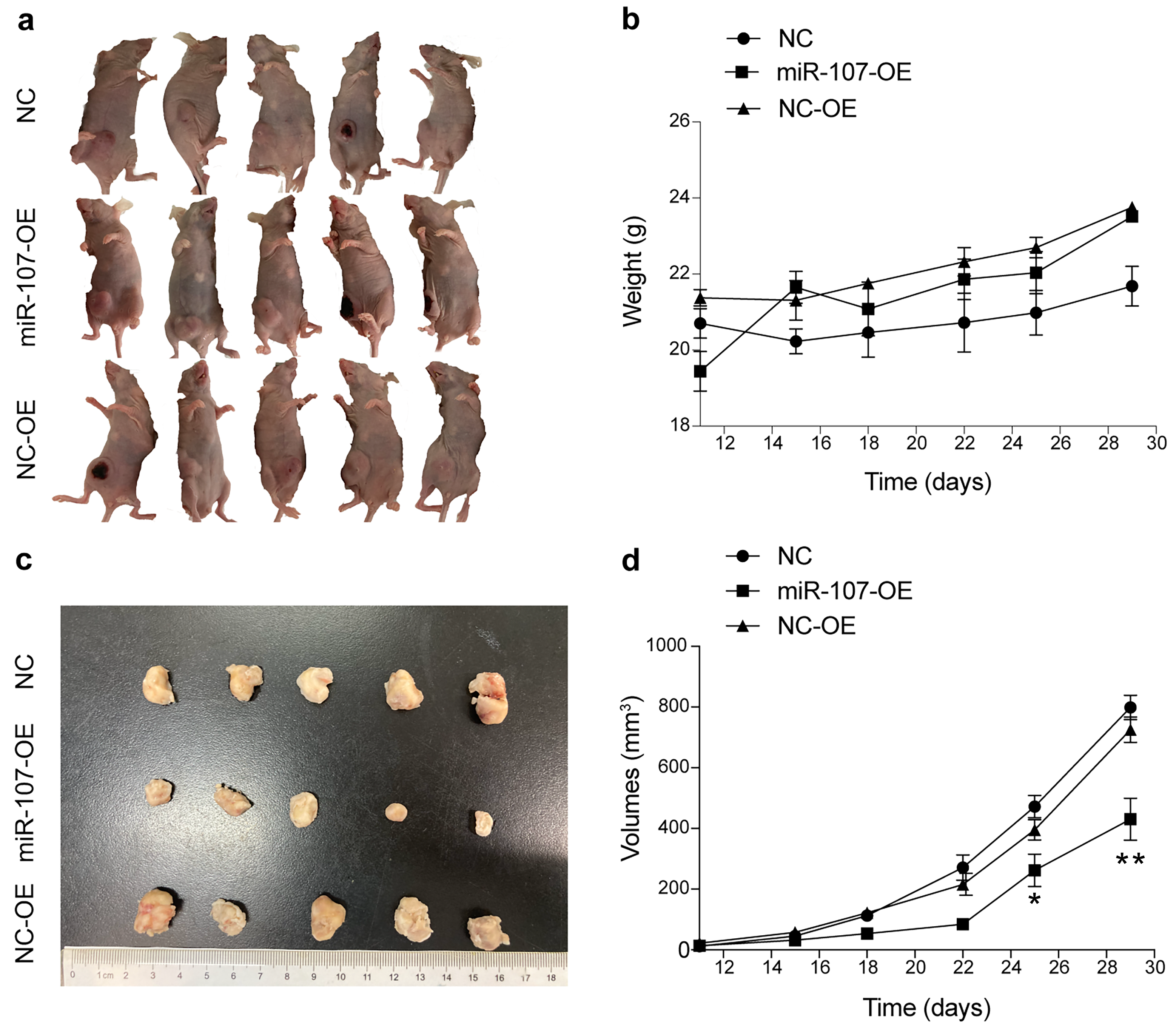

Supplement: Supplementary file 2 — Additional file 2: Supplemental Figure S2. miR-107 contributes to the inhibition of breast cancer development in an orthotopic breast cancer model. (a) Stably transfected MDA-MB-231 cells overexpressing miR-107 were injected into subcutaneous mammary fat pads of nude mice. Diagram of subcutaneous tumour formation in nude mice. (b) Body weight curve. (c, d) Tumour volume of xenograft tumours in different groups. NC, control group; miR-107-OE, miR-107 overexpression group; NC-OE, miR-107 empty group. n = 5; **p < 0.01. [file 12885_2022_9603_MOESM2_ESM.tif]
