# Supplementary material for: miR-107 is involved in the regulation of NEDD9-mediated invasion and metastasis in breast cancer
Source: BMC Cancer. 2022 May 12;22:533. doi: 10.1186/s12885-022-09603-3 (PMC9097419; doi:10.1186/s12885-022-09603-3)
Supplement: Supplementary file 3 — Additional file 3: Supplemental Table S1. The database for miRNA sequence-based prediction. [file 12885_2022_9603_MOESM3_ESM.docx]

**Supplementary Table 1** The database for miRNA sequence-based prediction

| **Type** | **Name** | **URL** |
| --- | --- | --- |
| Web-based | TargetScan | <http://www.targetscan.org/> |
|  | Diana Tools | <http://diana.imis.athena-innovation.gr/DianaTools/index.php> |
|  | miRanda | <http://www.microrna.org/microrna/getGeneForm.do> |
| Downloadable programs | miRanda | <http://www.microrna.org/microrna/getGeneForm.do> |
