# Supplementary material for: miR-107 is involved in the regulation of NEDD9-mediated invasion and metastasis in breast cancer
Source: BMC Cancer. 2022 May 12;22:533. doi: 10.1186/s12885-022-09603-3 (PMC9097419; doi:10.1186/s12885-022-09603-3)
Supplement: Supplementary file 4 — Additional file 4: Supplemental Table S2. Description of the parameters and values shown in the results delivered by TargetScan, miRanda and Diana Tools. [file 12885_2022_9603_MOESM4_ESM.docx]

**Supplementary Table 2** Description of the parameters and values shown in the results delivered by TargetScan, miRanda and Diana Tools.

| **Tools** | **Parameter** | **Value Range** | **Meaning** |  |
| --- | --- | --- | --- | --- |
| **TargetScan** | Site type | 8mer > 7mer > 7mer-A1 > 6mer | The matching sites in the seed region (nucleotides 2 to 8 from 5′ of miRNA that have perfect WC pairing with the 3′ UTR), from the strictest to the least strict. |  |
|  | Context++ score | From 1 to −1 | The sum of the contribution of 14 features for each of the four site types, the more negative the score, the greater the repression. |  |
|  | Context++ score percentile | From i to 100 − i; | Percentage of sites for the miRNA with a less favorable context++score. |  |
|  | Weighted context++ score | From 1 to −1 | The scores with a lower negative value indicate a greater prediction of repression. |  |
|  | Cumulative weighted context++ score | C(i–1) + (1 − 2CSi)(AIRi-C(i–1)) | This score estimates the total repression expected from multiple sites of the same miRNA, for each mRNA target predicted. |  |
|  | Branch-length score | 8mer: 1.8; 7mer-m8: 2.8; 7mer-A1: 3.6; 6mer: NA | This score is the sum of phylogenetic branch lengths between species that contain a matching site. |  |
|  | P_CT_ score | Between 0 and 1 | The higher the score, the greater the conservation and the greater mRNA destabilization expected. |  |
|  | Aggregate P_CT_ | Value = 1 − ((1 – P_CT_) site1 × (1 − P_CT_) site2 | For each miRNA, this parameter includes the conserved 3′ UTR targets with multiple sites that were missed in the human 3′ UTR annotation, but were present in the mouse annotations. |  |
|  | Conserved sites | ≥0 | Number of conserved sites identified. |  |
| **DIANA Tools** | miTG score | From 0 to 1 | This is a general score for the predicted interaction, the closer to 1, the greater the confidence. |  |
|  | Also Predicted | red, blue and green | This compares with other tools; miRanda in red, TargetScan in blue and TarBase in green. |  |
|  | Region | UTR3, CDS | Region of the mRNA where the interaction occurs. |  |
|  | Binding Type | 6mer; 7mer; 8mer; 9mer; miRNA bugle | The matching sites between the miRNA and the mRNA. |  |
|  | Score | From 0 to 1 | It is the site contribution score in the miTG score. |  |
|  | Conservation | ≥0 | Number of species in which the predicted interaction is conserved. |  |
|  | Signal-to-noise ratio (SNR) | >0 | This score is a measure of the “signal to noise” ratio, which enables the identification of the miTG score of each interaction without background noise. |  |
|  | Precision | From 0 to 1 | This score is an indicator of the false-positive rate in a miTG interaction. |  |
| **miRanda** | mirSVR score | <0 | This score is an estimate of the miRNA effect on the mRNA expression level. The more negative the score, the greater effect. |  |
|  | PhastCons score | From 0 to 1 | This measures the conservation of nucleotide positions across multiple vertebrates. |  |

**References:**

1. Riffo-Campos ÁL, Riquelme I, Brebi-Mieville P. Tools for Sequence-Based miRNA Target Prediction: What to Choose?. Int J Mol Sci. 2016;17(12):1987.
2. Witkos TM, Koscianska E, Krzyzosiak WJ. Practical Aspects of microRNA Target Prediction. Curr Mol Med. 2011;11(2):93-109.
