# Supplementary material for: miR-107 is involved in the regulation of NEDD9-mediated invasion and metastasis in breast cancer
Source: BMC Cancer. 2022 May 12;22:533. doi: 10.1186/s12885-022-09603-3 (PMC9097419; doi:10.1186/s12885-022-09603-3)
Supplement: Supplementary file 5 — Additional file 5. [file 12885_2022_9603_MOESM5_ESM.docx]

**Full original source data**

(including full scans of the entire original gels displayed in your manuscript, STR profiling data, the original files for any microscopy images included in your figures for each flow experiment with clear labels to differentiate separate experiments)

The download link is as follows:

<https://www.jianguoyun.com/p/Dc0Y-RsQlvWCChioqZwE>
